# Depression Dynamics Across a Decade: Daily Affective Experience and Yearly Depressive Symptomatology

**DOI:** 10.1093/geroni/igaa057.2052

**Published:** 2020-12-16

**Authors:** Raquael Joiner, C S Bergeman, Lijuan Wang, Guangjian Zhang, Kristin Valentino

**Affiliations:** 1 University of Notre Dame, Mishawaka, Indiana, United States; 2 University of Notre Dame, Notre Dame, Indiana, United States

## Abstract

Recent conceptualizations of depression and supporting empirical work suggests that elevations and allievations of depressive symptoms can be understood from a dynamic systems perspective. Specifically, depression is proposed to result from strong-feedback loops in a system comprised of highly interdependent component parts (e.g., affect states). Supporting this perspective, individual differences in emotional interia and strong connections across emotions at micro-level timescales have been consistently associated with individual differences in depressive symptomatology such that individuals with greater emotional inertia and cross-emotion relations show higher levels of depressive symptoms. Importantly, however, individual differences do not necessarily translate to intraindividual change. The present study explores whether emotional connectivity at the daily timescale differs within individuals across a ten-year span and how these associations relate to intraindividual changes in depressive symptomatology. The results of these individual-level analyses will help further a dynamic systems perspective of depression and help inform clinical interventions for depression.

